# Can We Retrieve the Information Which Was Intentionally Forgotten? Electrophysiological Correlates of Strategic Retrieval in Directed Forgetting

**DOI:** 10.3389/fpsyg.2017.01480

**Published:** 2017-08-29

**Authors:** Xinrui Mao, Mengxi Tian, Yi Liu, Bingcan Li, Yan Jin, Yanhong Wu, Chunyan Guo

**Affiliations:** ^1^Beijing Key Laboratory of Learning and Cognition, Department of Psychology, Capital Normal University Beijing, China; ^2^School of Psychological and Cognitive Sciences, Peking University Beijing, China; ^3^Beijing Advanced Innovation Center for Imaging Technology, Capital Normal University Beijing, China

**Keywords:** directed forgetting, strategic retrieval, ERP, retrieval inhibition, retrieval orientation

## Abstract

Retrieval inhibition hypothesis of directed forgetting effects assumed TBF (to-be-forgotten) items were not retrieved intentionally, while selective rehearsal hypothesis assumed the memory representation of retrieved TBF (to-be-forgotten) items was weaker than TBR (to-be-remembered) items. Previous studies indicated that directed forgetting effects of item-cueing method resulted from selective rehearsal at encoding, but the mechanism of retrieval inhibition that affected directed forgetting of TBF (to-be-forgotten) items was not clear. Strategic retrieval is a control process allowing the selective retrieval of target information, which includes retrieval orientation and strategic recollection. Retrieval orientation via the comparison of tasks refers to the specific form of processing resulted by retrieval efforts. Strategic recollection is the type of strategies to recollect studied items for the retrieval success of targets. Using a “directed forgetting” paradigm combined with a memory exclusion task, our investigation of strategic retrieval in directed forgetting assisted to explore how retrieval inhibition played a role on directed forgetting effects. When TBF items were targeted, retrieval orientation showed more positive ERPs to new items, indicating that TBF items demanded more retrieval efforts. The results of strategic recollection indicated that: (a) when TBR items were retrieval targets, late parietal old/new effects were only evoked by TBR items but not TBF items, indicating the retrieval inhibition of TBF items; (b) when TBF items were retrieval targets, the late parietal old/new effect were evoked by both TBR items and TBF items, indicating that strategic retrieval could overcome retrieval inhibition of TBF items. These findings suggested the modulation of strategic retrieval on retrieval inhibition of directed forgetting, supporting that directed forgetting effects were not only caused by selective rehearsal, but also retrieval inhibition.

## Introduction

Forgetting must be an efficient way to prevent irrelevant details from interfering with knowledge learning. Unfortunately, it is not always easy to figure out whether the forgotten information is worth remembering. Sometimes, during the examination, you might find a few questions hard to answer, because certain knowledge was intentionally ignored in learning. You realize that you have to use strategies to recall the useful information which you have directly forgotten. So, how do we retrieve the information which was intentionally forgotten?

Directed forgetting (DF) effects were demonstrated by lower memory performance of TBF items than TBR items (Van Hooff et al., 2009). In previous studies, item-cueing method was used to explore intentional forgetting in experiments (Hockley et al., 1998). At the study phase of item-cueing method, two explicit cues instructed subjects to remember the TBR (to-be-remembered) items and to forget TBF (to-be-forgotten) items respectively, following the presented items. At the test phase, TBR items were better recalled than TBF items, which is called DF effect of item-cueing method. This method of DF was involved with two hypotheses. The selective rehearsal hypothesis suggested that this effect is stemmed entirely from the diminished elaboration or rehearsal of TBF rather than TBR words at encoding phases (Levy and Anderson, 2008; Mecklinger et al., 2009; Xiao et al., 2012). That is, DF weakens the memory representation of TBF items at encoding phases, so that the difficulty of retrieval increased. Alternately, some ERP evidence suggested that learning instruction of DF blocked retrieval processes of TBF items, therefore supporting retrieval inhibition hypothesis (Hockley et al., 1998; Ullsperger et al., 2000; Racsmány and Conway, 2006; Van Hooff et al., 2009; Xiao et al., 2014). This means that the TBF items evoked cognitive control to inhibit retrieval processes, causing the low accuracy of TBF items.

According to the selective rehearsal hypothesis, some studies indicated that shallow encoding items caused the absence of late parietal old/new effect (Iidaka et al., 2006; Marzi and Viggiano, 2010). Curran (2004) found that late parietal old/new effects were reduced by divided attention, but early frontal old/new effects were not affected. One possible explanation is that the different patterns of retrieval between TBF items and shallow encoding items share the same memory process. However, Ullsperger et al. (2000) found that compared to shallowly encoded items, correctly recognized TBF words resulted in a qualitatively different pattern of the old/new effect. Recognition tests revealed that both deeply and shallowly encoded items elicited phasic frontal and parietal old/new effects, whereas TBF items showed less early frontal activity and the absence of the old/new effect at parietal sites. The retrieval processes of TBF items seemed to become inhibited, and less accessible, therefore, more difficult to retrieve. Thus, the current study explored whether the absence of late parietal old/new effect was solely due to weak memory encoding, or was caused by the additional role of retrieval inhibition processes when items followed by TBF instruction.

The investigation of strategic retrieval for TBF items could assist to explore whether the increased difficulty of TBF items was caused by inhibition retrieval processes. Strategic retrieval processes were defined as controlled processes, allowing the selective retrieval of information that was relevant to a specific situation and to the specific memory judgment (Moscovitch and Melo, 1997; Herron and Wilding, 2005). The exclusion memory task is a common paradigm to investigate strategic retrieval because subjects are required to identify target information and reject non-target information, forcing the use of strategic retrieval to retrieve more target information (Jacoby, 1991). In the exclusion task, subjects learned items in different (two or more) conditions at study phase. At test phase, subjects were asked to identify items of one target condition and to reject items of the other condition(s) as well as new items.

Results from recent ERP studies using the memory exclusion task suggested that strategic retrieval included two kinds of processes: retrieval orientation and strategic recollection (Rosburg et al., 2011a,b). Retrieval orientation is the specific form of processing which is applied to a retrieval cue when specific episodic information was targeted, and this process depends on retrieval difficulty (Rugg and Wilding, 2000). Comparing difficult retrieval tasks with simple tasks, difficult retrieval tasks demand more intentional efforts to complete memory search. The larger ERP differences between conditions were associated with higher levels of retrieval difficulty, which suggested that retrieval efforts modulated this retrieval orientation effect (Rosburg et al., 2011a). Therefore, the difference of retrieval orientation between these two tasks reflected the levels of retrieval efforts. Usually, studies on retrieval orientation focused on cortical responses to new items, because the processing of new items was assumed to be unaffected by retrieval success. The comparison between the tasks of old items is not only affected by the level of retrieval efforts but also affected by whether old items are retrieved successfully, because this comparison mixed memory trace with retrieval efforts. In summary, the comparison of new items avoid contamination of retrieval success.

On the other hand, the strategic recollection was defined as the controlled memory retrieval which strategically minimized the retrieval efforts to optimize the retrieval success (Rosburg et al., 2011b). Herron and Rugg (2003) pointed that strategic recollection was determined by the retrieval difficulty of target information. The strategic recollection consists of two strategies: recall-to-reject strategy and task-specific strategy.

Recall-to-reject strategy is the retrieval of non-target source information that is potentially beneficial, because it could promote a swift rejection decision for non-target information in a memory exclusion task (Clark, 1992). When target accuracy was low (indicating difficult retrieval), subjects tended to use the recall-to-reject strategy. The retrieval of non-target information was resulted by difficult retrieval of target information, because non-target information could provide more reliable information for classifying items as targets and non-targets (Dzulkifli and Wilding, 2005; Wilding et al., 2005; Dzulkifli et al., 2006).

Alternatively, the task-specific retrieval strategy prevented the retrieval of non-target information in order to enhance the retrieval processes of specific target information. That is, subjects might endorse a source-specifying item as the target and reject all other available items (Herron and Rugg, 2003). When target accuracy was high (indicating easy retrieval), subjects tended to use the task-specific retrieval strategy. This retrieval strategy focuses on target information and is an efficient way for subjects to identity target items, because low retrieval difficulty target information easily captures cognitive resources, and the rejection of all other items avoided the interference of unrelated information.

Such strategic control processes in retrieving target information might be involved in overcoming memory interference, demonstrating that strategic retrieval benefit for the retrieval processes of target information (Bergström et al., 2012). As control processes of retrieval, strategic retrieval may affect the retrieval processes of TBF items rather than memory representation. Retrieval inhibition of DF was a process to suppress retrieval of TBF items and to selectively retrieve TBR items. In contrast, when TBF items were retrieval target, TBF items which should have been inhibited in retrieval process were retrieved selectively. Therefore, the top–down control of strategic retrieval converts TBF items into retrieval targets and overcome their retrieval inhibition.

Recognition retrieval was involved with the early frontal old/new effect and the late parietal old/new effect. Based on dual-process framework, recognition memory includes two processes—familiarity which is a fast and automatic process underpinning a general feeling of prior occurrence, and recollection which is a slower process supporting conscious retrieval of specific episodic details (Yonelinas, 2002). The early frontal old/new effect (a positive shift or reduction in negativity in frontal regions at 300∼500 ms) indexed familiarity and the late parietal old/new effect (a positive component in posterior regions at 500∼800 ms) indexed recollection (Curran, 2000; Curran and Hancock, 2007; Diana et al., 2007; Rugg and Curran, 2007).

Normally, strategic recollection is associated with the late parietal old/new effect: this old/new effect of non-targets was smaller than the effect of targets, when task-specific strategy was used; this old/new effect of non-targets was the same as the effect of targets, when recall-to-reject strategy was used (Herron and Rugg, 2003; Wilding et al., 2005; Rosburg et al., 2011b). In the process of strategic retrieval, the late parietal old/new effect in response to targets is considered a reliable measurement for strategic recollection. For retrieval orientation, ERPs of new items were more positive than old items from 600 to 1100 ms when items of the difficult retrieval were targeted (Rosburg et al., 2011a).

Our current study aimed at clarifying the performance of strategic retrieval in item-method DF paradigm. The first goal of our study was to explore whether TBF items could be strategically retrieved, which assisted in proving the hypothesis of retrieval inhibition. If TBF items were retrieval targets and they evoked no ERP old/new effects, then, DF effects might be elicited by weak encoding representation alone. In that case, the memory strength of TBF items would be too weak to retrieve the items, thus supporting that DF effects in item method were only based on selective rehearsal hypothesis. If TBF items were retrieval targets and they could elicit ERP old/new effects, DF effects might be elicited by retrieval inhibition. Strategic retrieval could alleviate inhibition to reactivate memory representation of TBF items, therefore supporting that the retrieval inhibition hypothesis also explains the DF effects.

The second aim of our study was to investigate the mechanism of strategic retrieval (including retrieval orientation and strategic recollection) for TBF items, and if they could be strategically retrieved. Previous evidence indicated that the difficulty of retrieval modulated the potential impact of retrieval efforts on retrieval orientation (Rosburg et al., 2014). Since the difficulty of target retrieval was enhanced by DF instruction, participants would retrieved TBR targets more accurately than TBF targets. Therefore, we hypothesized that ERPs to new items were more positive when TBF items were targeted than when TBR items were targeted. This correlation of retrieval orientation reflected that TBF items which were targeted demanded more efforts to retrieval. For strategic recollection, we hypothesized that TBR items, but not TBF items, would be retrieved as non-targets, because the retrieval of TBR items was expected to be easier than the retrieval of TBF items, which would elicit the recall-to-reject strategy in the current design. We also predicted that when TBF items which were targeted (vs. TBR items which were targeted), strategic recollection evoked the late parietal old/new effects for both target and non-target retrieval.

## Materials and Methods

### Participants

Twenty one paid volunteers participated in the study, all were native Chinese students (13 women and 8 men) aged 20–30 years (mean age, 23.8 years) from Capital Normal University (Beijing, China). All subjects were right-handed, with normal or corrected to normal vision, and no reported history of psychiatric or neurological disorders, head injury, or psychotropic drug use. Only one male participant was excluded due to excessive artifacts (artifact-free ERP trials were less than 18 in some conditions), leaving a final sample of 20 participants. Each subject signed an informed consent form for the experimental protocol, which was approved by the Capital Normal University Human Research Committee.

### Materials

Two-character Chinese nouns (360 in total) were used as stimuli [mean total number of strokes: 16.51 (ranging from 5 to 35), mean word frequency: 16.50 (ranging from 2.3 to 99.7) occurrences per million words (Liu et al., 1990)]. Another fifteen adult native Chinese speakers (an independent sample; seven men) rated the concreteness of these nouns. The concreteness ratings (from 1/extremely abstract to 7/extremely concrete) confirmed that the set of nouns was concrete nouns (Mean = 6.33, ranging from 5.38 to 6.92). All 360 nouns were randomly separated into three equal sets (120 nouns for TBR words, 120 nouns for TBF words and 120 nouns for unstudied words). The 120 TBR words and 120 TBF words were used as “old” (studied), and the other 120 nouns were used as “new” (unstudied) items at the test. The items of the three sets were randomly arranged to have equivalent concreteness, number of strokes or word frequency. There were four study blocks and four test blocks in each session. Two test blocks were designed to use TBR words as retrieval targets (TBR_T condition), the other two test blocks were designed to use TBF words as retrieval targets (TBF_T condition). The order of test conditions was counterbalanced across individuals (test blocks and study blocks were correlated). In each study phase, there were 30 TBR words and 30 TBF words. In each of the test phrases, there were 30 targets to be identified, and 30 old items of non-targets, together with 30 new items that had to be rejected.

### Procedure

**FIGURE 1:**
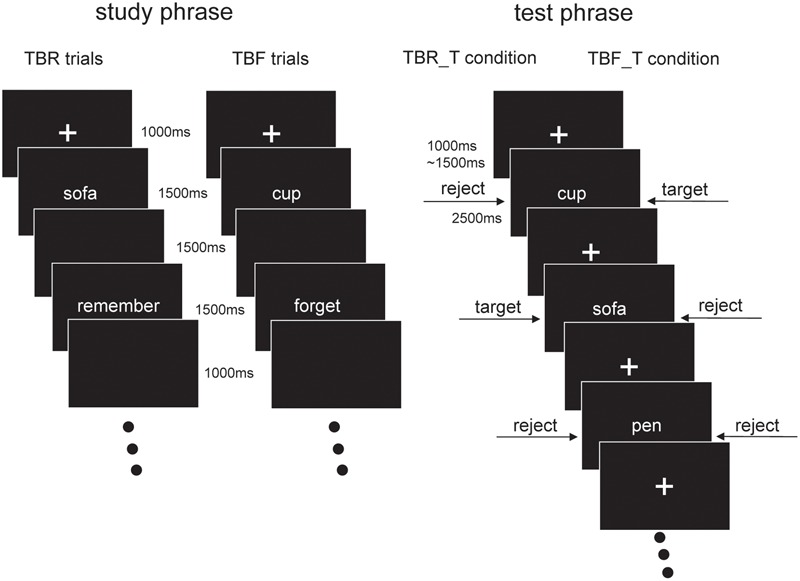
Experimental paradigm. In our experiment, participants completed a directed forgetting paradigm at study phase, as well as a memory exclusion paradigm at test phase.

The present study used a DF paradigm as well as a memory exclusion task. Item-cueing method, a typical method of “DF paradigm,” which presented the cue following each item, was used in our experiment. Participants were seated 75 cm from a Dell monitor in an electrically shielded room wherein they performed the experimental tasks. After a short practice block, participants undertook the experiment, which consisted of four study blocks and four test blocks, all four blocks of study phase were followed by four blocks of test phase. There was a 2 min rest period between study blocks or between test blocks, while between study and test blocks, there was a 5 min rest period. Four study blocks were presented at first. During the study phase, subjects did not know the instructions of following tests. Two of test blocks belonged to TBR item target (TBR_T) condition; the other two blocks belonged to TBF item target (TBF_T) condition. Subjects were tested with blocks of TBR_T before TBF_T, in order to prevent from trying to remember the TBF items. An EEG was recorded throughout the sessions (**Figure 1**). During the study phase, each trial began with a fixation cross (1000 ms) that was followed by a noun (extending a 3.51° × 1.83° visual area), which was centrally presented for 1500 ms, then an empty screen appeared for 1500 ms. After that, instruction (TBR vs. TBF) was presented for 1500ms, and ended up with an empty screen for 1000 ms. All stimuli were presented in white against a black background. The order of trials was pseudo-random with each type of instruction (TBR vs. TBF) appearing in no more than three consecutive trials. Participants were explicitly instructed to follow the instructions to either remember the nouns or to forget them. After the study phase had been completed, subjects were informed of the testing instruction. At the test, participants were tested in a memory exclusion task with the target category switching after half of the blocks: in the TBR item target (TBR_T) condition, participants had to identify the TBR words and to reject TBF words together with new words. In the TBF item target (TBF_T) condition, participants had to identify TBF words and to reject TBR words together with new words. As illustrated in **Figure 1**, noun presentation did not differ between the two test conditions. Participants were instructed to respond as fast and accurately as possible. In the test phase, trials started with a fixation cross, lasting for 1000–1500 ms. Then, the tested items were presented for 2500 ms. Participants responded by pressing the letters “F” and “J” on a computer keyboard with the left and right index finger. The assignment of the key to the response category (Targets vs. Non-targets) was balanced across participants. The whole experiment took about 2.5 h (including preparation time for EEG recording).

### ERP Recording and Analyses

For each test condition, the discrimination index (Pr) was quantified as the difference between the hit rate (P_target) and the false alarm rate to non-targets (P_false alarm; Snodgrass and Corwin, 1988), with TBR_T and TBF_T test conditions. Behavioral responses were compared between the two conditions by means of paired *t*-tests and repeated measure analysis of variance (ANOVA). Target items given target responses were deemed to be “target hit” responses; non-target items given non-target responses were considered “non-target hit” responses; and new items given non-target responses were considered “correctly rejection.” Target items given non-target responses were deemed to be “miss” responses; non-target items given target responses were considered “non-target false alarm” responses; and new items given target responses were considered “target false alarm.” The hit rate was calculated as the ratio of the number of hit over its number of items, and the false alarm rate was calculated as the ratio of the number of false alarm over its number of items (Mollison and Curran, 2012). TBR items was considered as retrieval targets for the TBR_T test conditions, and TBF items was considered as retrieval targets for TBF_T test conditions. The data of RTs was log transformed to analyze.

In order to analyze strategic recollection, old/new effects for targets and non-targets were compared between the two conditions. In the TBR_T condition, old/new effects of TBR items which were identified as targets were compared with those of TBF items that were identified as non-targets. In the TBF_T condition, old/new effects of TBF items which were identified as non-targets were compared with those of TBR non-targets. Mean ERP amplitudes were extracted from two time windows (300∼450, 450∼650 ms after test item onset) to estimate the old–new effect as indexed by the early frontal old/new effect and late parietal old/new effect. The time windows were selected based on both visual inspection of the grand average ERP waveform and previous ERP literatures on familiarity (early frontal old/new effect) and recollection (late parietal old/new effect; Rugg and Curran, 2007). Electrodes were selected *a priori* to form two regions of interest (ROIs) centered around midline frontal and parietal sites that best capture early frontal old/new effects and late parietal old-new effects, with early frontal old/new effects for anterior sites and late parietal old/new effects for posterior sites (Anterior sites: F3, F4, Fz, FC3, FC4, FCz; Posterior sites: P3, P4, Pz, PO3, PO4, POz). In order to explore the retrieval orientation effect, ERPs to new items were contrasted between the TBR_T and TBF_T conditions. Based on previous ERP studies of retrieval orientation (Rosburg et al., 2013), we focused on the time window between 700 and 900 ms. In order to assess the topography of the retrieval orientation effect, ERP data of new items were entered in an ANOVA with Condition (TBR_T vs. TBF_T), Location (Anterior vs. Posterior), as within subject factors. The Greenhouse–Geisser adjustment for non-sphericity was used when necessary, as indicated by reporting the corrected *p*-values together with the uncorrected degrees of freedom.

EEG was recorded from a 62-channel Neuroscan system at 500 Hz sampling rate with a 0.05–100 Hz bandpass filter. Electrooculogram (EOG) was recorded at two eye electrodes at the outer canthi of each eye and one infraorbital to the left eye. EEG signals were referenced to the left mastoid during recording and re-referenced offline to the average of the left and right mastoid recordings. EEG/EOG signals (impedance < 5 kΩ) were digital bandpass filtered from 0.05 to 40 Hz, segmented around image onset (-100 ∼1000 ms) and corrected to a 100 ms pre-stimulus baseline. Trials with EEG voltages exceeding ± 75 μV were excluded from analysis. EOG blink artifacts were corrected using a linear regression estimate. Experiment presentation was executed using Presentation (Neurobehavioral Systems, Inc). Data collection was performed using Neuroscan acquisition software, and statistical analysis was performed in SPSS 20.0.

## Results

### Behavioral Data

The analysis of the behavioral data of the test phase revealed a higher hit rate for targets in the TBR_T condition [TBR_T -TBF_T = 0.09 (0.03); *t*(19) = 2.87, *p* < 0.05], with higher rates of false alarms for targets in the TBF_T condition [TBR_T -TBF_T = -0.13 (0.04); *t*(19) = -3.37, *p* < 0.01]. As a consequence, the discrimination index (Pr) was higher for the TBR_T condition than the TBF_T condition [TBR_T -TBF_T = 0.21 (0.04); *t*(19) = 5.23, *p* < 0.001]; thus, participants are demonstrated with a liable DF effect. In addition, the percentage of correctly rejected new items was higher when TBR items were targeted [TBR_T -TBF_T = 0.12 (0.04); *t*(19) = 3.24, *p* < 0.01].

The RTs (reaction times) differed between test conditions for targets and new items of correct responses, with faster responses for the TBR_T condition than the TBF_T condition [targets: TBF_T -TBR_T = 0.09 (0.08); new items: TBF_T -TBR_T = 0.54 (0.48); *t*(19) > 4.90, *p* < 0.001]. In the TBR_T condition, responses were fastest for new items than both non-targets and targets [non-target – new = 0.11(0.05); target – new = 0.08 (0.04); *t*(19) > 9.03, *p*s < 0.001]. In the TBF_T condition, responses were also fastest for new items, than both non-targets and targets [non-target – new = -0.37 (0.46); target- new = -0.41 (0.51); *t*(19) > -3.64, *p*s < 0.01], but there was no significant difference between targets and non-targets [target – non-target = 0.03 (0.13); *t*(19) = 1.01, *p* = 0.29]. In sum, lower retrieval accuracy and slower response times for the retrieval of TBF_T targets reflected that participants had more difficulties in retrieving TBF_T targets (see **Table 1**).

**Table 1:** Behavioral results.

	Recognition rate		Reaction time	
	TBR_T	TBF_T	TBR_T	TBF_T
Hit_target	0.76(0.09)	0.67(0.13)	1069.21(166.10)	1333.42(244.87)
Hit_non-target	0.78(0.12)	0.66(0.18)	1162.60(216.13)	1322.54(395.19)
Correct rejection	0.95(0.09)	0.83(0.20)	893.66(153.60)	1035.45(292.44)
Pr	0.54(0.18)	0.34(0.26)		
Miss	0.24(0.08)	0.32(0.13)	1182.29(244.87)	1318.10(401.62)
Fal_non-target	0.22(0.12)	0.34(0.18)	1266.18(344.24)	1370.20(377.81)
Fal_target	0.04(0.08)	0.17(0.20)	699.93(493.13)	1185.79(496.12)

*^∗^Standard deviations in parentheses. Hit_target, “target hit”; Hit_non-target, “non-target hit”; Fal_non-target, “non-target false alarm”; Fal_target, “target false alarm.” The RT values are raw data.*

### ERP Data

**FIGURE 2:**
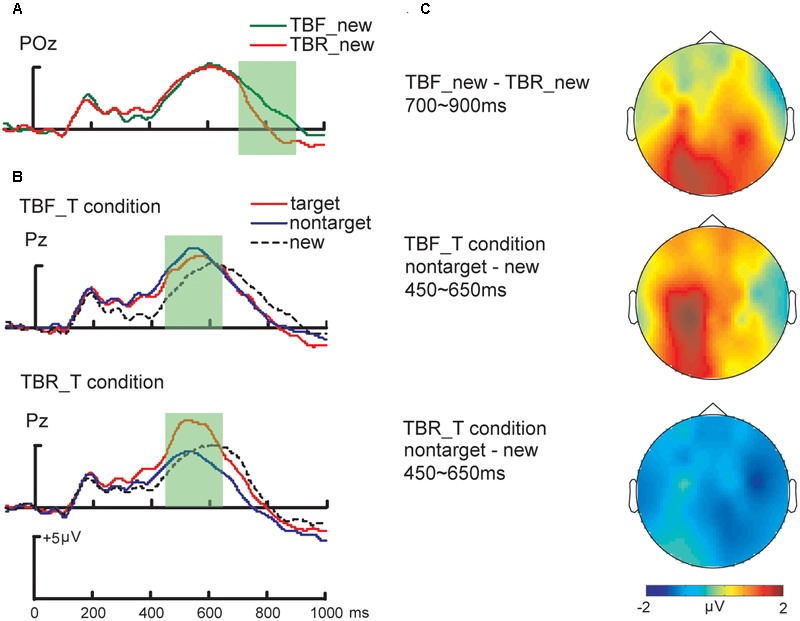
ERPs of strategic retrieval. **(A)** ERPs to new items in the two target conditions: data from the POz electrode are shown; ERPs to new items in the TBR_T condition are plotted as a red line, ERPs to new items in the TBF_T condition as a green line. **(B)** The late parietal old/new effects for targets, non-targets and new items at the electrode Pz are shown, separately for the TBR_T and TBF_T condition. **(C)** The topographic maps of retrieval orientation showed that amplitudes of TBR (to be remembered) instruction were more positive than that of TBF (to-be-forgotten) instruction during 700∼900 ms time windows. The topographic maps of strategic recollection showed that TBR (to-be-remembered) instruction diminished differential amplitudes between non-target and new items during time windows of late parietal old/new effects (450∼650 ms).

In order to assess the retrieval orientation effect, ERP data were entered in a repeated ANOVA with Location (Anterior vs. Posterior), and Condition (TBR_T vs. TBF_T) as within subject factors. Comparing ERPs to new items between the TBR_T and TBF_T conditions, we focused on 300∼450 ms, 450∼650 ms, and 700∼900 ms time windows. In 300∼450 ms time window, the repeated ANOVA with factors of Location (Anterior vs. Posterior) and Condition (TBR_T vs. TBF_T) revealed no significant main effect [*F*(1,19) < 3.65, *p*s > 0.07, $\eta_{p}^{2}$ = 0.17], or no significant two-way interaction [*F*(1,19) = 1.18, *p* = 0.29, $\eta_{p}^{2}$ = 0.58]. For 450∼650 ms time window, a repeated-measures ANOVA with factors of Location (Anterior vs. Posterior) and Condition (TBR_T vs. TBF_T) revealed a significant main effect of location [*F*(1,19) = 16.86, *p* = 0.001, $\eta_{p}^{2}$ = 0.47], with no significant main effect of condition [*F*(1,19) = 0.12, *p* = 0.73, $\eta_{p}^{2}$ = 0.01] or significant interaction between the two variables [*F*(1,19) = 1.55, *p* = 0.23, $\eta_{p}^{2}$ = 0.08]. For 700∼900 ms time window, a significant interaction between location and retrieval orientation was observed [*F*(1,19) = 14.73, *p* < 0.001, $\eta_{p}^{2}$ = 0.44]. In posterior sites, ERP to new items (700∼900 ms) were found to be more positive for the TBF_T condition than the TBR_T condition [TBF_T -TBR_T = 1.52 (0.61) μV; *t*(19) = 2.51, *p* < 0.05; **Figure 2A**]; but in anterior sites, there was no difference between TBF and TBR condition [TBF_T -TBR_T = 0.25 (0.48) μV; *t*(19) = 0.52, *p* = 0.61]. The findings on retrieval orientation showed that ERPs to new items were more positive-going for the TBF_T condition. This effect had the same polarity and a similar time course as in the previous study, but was topographically more posterior (Rosburg et al., 2011a; **Figure 2C**).

We then analyzed ERP old/new effects (early frontal old/new effects and late parietal old/new effects) as critical neural indices of strategic retrieval. For early frontal old/new effects (300∼450 ms) at the anterior site, a repeated-measures ANOVA with factors of Condition (TBR_T vs. TBF_T) and Stimulus (Target vs. Non-target vs. New) revealed a significant main effect of stimulus [*F*(2,38) = 14.56, *p* < 0.001, $\eta_{p}^{2}$ = 0.43], with no significant main effect of condition [*F*(1,19) = 0.15, *p* = 0.70, $\eta_{p}^{2}$ = 0.08] or significant interaction between the two variables [*F*(2,38) = 2.04, *p* = 0.14, $\eta_{p}^{2}$ = 0.10]. The stimulus effect indicated that the early frontal old/new effect for target was more positive than both non-target and new trials [target – non-target = 1.07 (0.29) μV; target – new = 1.31 (0.28) μV; *t*(19) > 3.69, *p*s < 0.01], but there was no difference between non-target and new trials [non-target – new = 0.24 (0.20) μV; *t*(19) = 1.21, *p* = 0.24]. For late parietal old/new effect amplitudes (450∼650 ms) at the posterior site, the repeated ANOVA with factors of Condition (TBR_T vs. TBF_T) and Stimulus (Target vs. Non-target vs. New) revealed a significant main effect of stimulus [*F*(1,19) = 9.02, *p* < 0.001, $\eta_{p}^{2}$ = 0.32] and a significant interaction [*F*(2,38) = 7.12, *p* < 0.01, $\eta_{p}^{2}$ = 0.27], but no significant main effect of condition [*F*(1,19) = 0.49, *p* = 0.50, $\eta_{p}^{2}$ = 0.03]. The main effect of stimulus suggested that the late parietal old/new effects for targets were more positive than non-target and new trials [target- new = 1.37 (0.33) μV; target – non-target = 1.10 (0.39) μV; *t*(19) > 2.85, *p*s < 0.05], but there was no difference between non-target and new trials [non-target – new = 0.27 (0.31) μV; *t*(19) = 0.88, *p* = 0.39]. In the TBR_T condition, the late parietal old/new effect to targets were more positive than to non-targets and new items [target – non-target = 2.20 (0.59) μV; target – new = 1.67 (0.50) μV; *t*(19) > 3.35, *p*s < 0.01; **Figure 2B**], with no difference between the latter two [non-target – new = -0.53 (0.39) μV; *t*(19) = -1.37, *p* = 0.19; **Figure 2B**].

However, for the TBF_T condition, the left-parietal ERPs to targets and non-targets were more positive than to new items [target – new = 1.07 (0.27) μV; non-target – new = 1.08 (0.42) μV; *t*(19) > 2.57, *p*s < 0.05; **Figure 2B**], but there was no difference between target and non-target trials [target – non-target = -0.09 (0.50) μV; *t*(19) = -0.02, *p* = 0.99; **Figure 2B**]. In 700∼900 ms time window, the repeated ANOVA with factors of Location (Anterior vs. Positerior), Condition (TBR_T vs. TBF_T) and Stimulus (Target vs. Non-target vs. New) revealed no significant main effect [*F*(1,19) < 3.20, *p*s > 0.05, $\eta_{p}^{2}$ = 0.15], or no significant three-way interaction [*F*(2,38) = 0.15, *p* = 0.86, $\eta_{p}^{2}$ = 0.008]. These findings suggested that the ERP difference between TBR_T and TBF_T condition was observed in 450∼650 ms time window of posterior sites. When TBF items were considered as targets, old/new effects were elicited by both target and non-target items. When TBR items were considered as targets, only target items (TBR items) could evoke old/new effects.

## Discussion

As for behavioral data, our participants exhibited retrieval advantages for the TBR targets, with more accurate and faster retrieval of TBR targets than that of TBF targets, which is similar as previous studies of DF tasks (Sahakyan and Kelley, 2002; Wylie et al., 2008). The relatively poor performance of TBF targets might indicate more difficult retrieval for TBF items. Given that retrieval RTs were found to be modulated by the retrieval orientation effect, the retrieval RTs of new items were faster for TBR_T condition than for TBF_T condition.

According to dual-process models of recognition memory, the early frontal old/new effect was believed to reflect familiarity-related processes, and the late parietal old/new effect was suggested to index recollection-related processes (Rugg and Curran, 2007). Familiarity was often operationally defined as recognition processes without retrieving details of events, which was sensitive to memory strength. In the current study, an early frontal old/new effect was observed in response to target items independently of instructions (TBR vs. TBF). Under both TBR and TBF instruction, the targets were recognized successfully without any contextual details. When the TBR items were targets, they were easy to recognize, because their memory strength were higher than TBF items. Although TBF items were difficult to retrieve intentionally, subjects still recognized the TBF items successfully, without retrieving non-targets of TBR items to promote the familiarity. These findings suggested that familiarity could be modulated by top–down processes of strategic retrieval and therefore indicating that the memory representation was strong enough to intentional retrieval, especially for TBF items.

In addition, recollection was defined as recognition processes with contextual details retrieved. Previous ERP evidences of DF effects on retrieval was accompanied by the absence of late parietal old/new effects (Ullsperger et al., 2000). This means that the absence of late parietal old/new effects was an index of retrieval inhibition and this retrieval inhibition repressed the process of recollection. However, our ERP results suggested that TBF items which were retrieval targets could elicit late parietal old/new effects, suggesting that strategic retrieval could alleviate retrieval inhibition to reactivate recollection of TBF items representation. When the TBR items were targets, the TBF items were not recalled; when TBF items were targets, the TBF items were successfully recalled. The different retrieval targets changed the effects of retrieval inhibition to influence the retrieval success, which further suggested the impact of retrieval inhibition on DF effects. Therefore, the impaired retrieval elicited by DF instruction may not only be due to weak encoding representation, but also by retrieval inhibition.

The mechanism of strategic retrieval included strategic recollection and retrieval orientation. The investigation of strategic retrieval for TBF items helped clarify how strategic retrieval could overcome retrieval inhibition. Our ERP results of retrieval orientation effects were examined to compare TBR_T condition with TBF_T condition. The previous studies of retrieval orientation showed that ERPs to new items were more positive at frontal electrode sites between 600 and 1100 ms when difficult retrieval items were targeted (vs. easy retrieval items were targeted; Rosburg et al., 2011a). This effect of test condition on the processing of new items was interpreted as a retrieval orientation effect, due to the higher level of retrieval efforts for difficult target-retrieval conditions. In line with this argument, our study revealed more positive ERPs to new items when TBF items were targeted (vs. when TBR items were targeted), supporting our hypothesis that the correlation of retrieval orientation reflected that retrieval tasks for TBF targets demanded more retrieval efforts to overcome retrieval inhibition. The retrieval orientation effects in pervious and the present study were similar in their temporal characteristics (700∼900 ms time window), in which their topographic distributions differed between the studies. In our currently study, the retrieval orientation effect was found exclusively at posterior electrode sites, while previous topography of the observed effects was found in frontal electrode sites extending to posterior electrode sites. The topography of a retrieval orientation effect could be assumed to be dependent on retrieval efforts of different information (Rosburg et al., 2011a). Previous research indicated that the maintenance of memory retrieval was thought to be governed by a cortical-basal ganglia-thalamo-cortical loop (Depue, 2012). In this loop, sensory representations in posterior cortex are actively maintained by retrieval control. Thus, TBF instructions might elicit more retrieval efforts and modulate the topography of a retrieval orientation effect that was observed at posterior electrode sites (Dzulkifli and Wilding, 2005; Mecklinger, 2010).

Also, strategic recollection was examined to investigate which strategy was used in the processes of strategic retrieval. As previous studies mentioned, there were two types of strategic recollection: the recall-to-reject strategy was the retrieval of non-targets information to reject non-targets, while the task-specific retrieval strategy would trend to retrieve specific targets rather than non-target items (Herron and Rugg, 2003). The non-target retrieval was suggested to be probably governed by the retrieval difficulty of target information (Herron and Rugg, 2003) or the type of strategic recollection which occurred relied on the retrieval difficulty of targets. Our behavioral results exhibited more accurate and faster retrieval for TBR targets than TBF targets, suggesting that TBF targets were more difficult to retrieve. Since the TBF instruction resulted in less elaborative rehearsal and more retrieval inhibition for TBF items, DF was suggested to increase the retrieval difficulty of TBF items. We used the late parietal old/new effect as a reliable measurement for strategic recollection, because the late parietal old/new effect was demonstrated to be associated with strategic recollection (Herron and Rugg, 2003; Wilding et al., 2005; Rosburg et al., 2011b). When TBR items were considered as targets, the retrieval difficulty of targets was low and the retrieval task was retrieving targets rather than non-targets, thus reflecting the engagement of task-specific retrieval processes modulating what is retrieved (Dzulkifli and Wilding, 2005). Our ERP evidence showed that the late parietal old/new effect was only evoked by targets (TBR items), reflecting the task-specific retrieval strategy which was beneficial for retrieval inhibition of TBF items, in order to selectively retrieve target information. This retrieval strategy is an efficient way to avoid the disturbing of unrelated information and to identify the target information of low retrieval difficulty.

However, the possibility of recall-to-reject strategy was demonstrated to increase with increased retrieval difficulty. The recall-to-reject retrieval strategy was shown to occur when target accuracy was lowered by increasing the task difficulty (Dzulkifli et al., 2006). In this type of task, retrieval of non-target source information is potentially beneficial, as it promotes a swift rejection decision for non-targets (Rosburg et al., 2013). Our results of frontal old/new effects showed that the non-targets (TBR items) did not evoke frontal old/new effects when TBF items were considered as targets, suggesting that the memory trace of TBF items provided a plenty of information to elicit familiarity. However, the processes of familiarity were not involved with contextual information which were necessary to decide whether the items were targets or non-targets. Therefore, strategic retrieval was reflected in the processes of recollection which was retrieval of details. We found late parietal old/new effects of non-targets only in the more difficult retrieval condition. When TBF items were considered as targets and TBR items were considered as non-targets, the late parietal old/new effect was evoked by non-targets (TBR items) and targets (TBF items), reflecting recall-to-reject strategy. Due to the difficult retrieval of target items, the target information which was retrieved was hard to provide plentiful contextual information for identifying target items. In order to identify TBF item and reject TBR item, non-targets information were retrieved to promote more accurate decision-making.

## Conclusion

In summary, we found that DF influenced strategic retrieval and demonstrated that DF effects in item-cueing method was caused by both selective rehearsal and retrieval inhibition. Retrieval orientation effects of DF showed that new items of TBF_T condition elicited more positive amplitudes than new items of TBR_T condition, which proved that TBF item demanded more retrieval efforts. Additionally, the type of strategic recollection relied on DF: (a) when TBR items were considered as the target, recollection (late parietal old/new effect) was only evoked by TBR items, reflecting task-specific retrieval strategy which was benefit for inhibit retrieval of TBF items; (b) when TBF items were considered as targets, recollection (late parietal old/new effect) was evoked by both TBR items and TBF items, reflecting recall-to-reject strategy which promoted more accurate decision-making to overcome retrieval inhibition.

## Ethics Statement

Each subject signed an informed consent form before experiment and received monetary compensation after experiment. This study was carried out in accordance with the recommendations of “Human Research Ethics Committee at Capital Normal University”; with written informed consent from all subjects. All subjects gave written informed consent in accordance with the Declaration of Helsinki. The protocol was approved by the “Human Research Ethics Committee at Capital Normal University.” No additional considerations of the study in cases where vulnerable populations were involved.

## Author Contributions

CG supervised the project and designed the study. MT collected and analyzed the data. XM wrote the main manuscript text and prepared **Figures 1, 2**. YL, BL, and YW revised the draft of manuscript. YJ took the charge of language revision. All authors reviewed the manuscript.

## Conflict of Interest Statement

The authors declare that the research was conducted in the absence of any commercial or financial relationships that could be construed as a potential conflict of interest.
